# Feasibility of Breast MRI as the Primary Imaging Modality in a Large Asian Cohort

**DOI:** 10.7759/cureus.15095

**Published:** 2021-05-18

**Authors:** Fang-Ying Li, Alan Hollingsworth, Wai-Tak Lai, Tsung-Lung Yang, Liang-Juan Chen, Wei-Teng Wang, Jing-Lung Wang, Abraham N Morse

**Affiliations:** 1 Administration, Taitung Saint Mary's Hospital, Taitung, TWN; 2 Medical Affairs, Aurora Healthcare United States Corporation, Oklahoma City, USA; 3 Research and Development, Aurora Healthcare Asia, Taipei, TWN; 4 Innovation, Kaohsiung Veterans General Hospital, Kaohsiung, TWN; 5 Quality Management Center, Kaohsiung Veterans General Hospital, Kaohsiung, TWN; 6 Radiology, Kaohsiung Veterans General Hospital, Kaohsiung, TWN; 7 Radiology, Taitung Saint Mary's Hospital, Taitung, TWN; 8 Medical Affairs, Aurora Healthcare United States Corporation, Danvers, USA; 9 Obstetrics and Gynecology, Tufts University Medical Center, Boston, USA

**Keywords:** breast mri, breast cancer screening, breast cancer, cancer detection rate, positive predictive value, breast density, asia, false positive rate

## Abstract

Purpose

Contrast-enhanced MRI has repeatedly demonstrated significantly enhanced sensitivity compared to mammography and ultrasound in breast cancer detection. The purpose of this study was to evaluate the feasibility and outcomes of using breast MRI as the initial imaging study for screening and diagnosis.

Materials and methods

In this retrospective review of a cohort of 10,374 breast MRI scans in 7967 patients in Taitung County, Taiwan, a total of 5619 participants met inclusion criteria and were included in our analysis. We reviewed all biopsies that were performed subsequent to MRI studies in women (screening vs. diagnostic). The primary outcomes were false-positive (FP) biopsy rates and positive predictive value (PPV) of MRI - parameters that have historically been associated with performance that restricts more widespread use of MRI. False-positive rate based on benign biopsies (FPR-3) and the positive predictive value (PPV-3) were calculated.

Results

Without complementary imaging or follow-up to identify false negatives, the study of performance characteristics was limited to false positives and PPV. There were 351 benign biopsies generated by MRI out of the cohort of 5555 participants (5619 minus the malignant biopsies), generating a false-positive rate of 6.3%. Sixty-four patients out of 415 biopsies were malignant, generating a PPV-3 of 15.4%.

Conclusion

In this Asian cohort, utilizing breast MRI as the initial study for screening and/or diagnosis appears to be limited more by practical considerations such as cost and patient flow efficiency than by feasibility based on performance characteristics. With well-established superior sensitivity, coupled with improved interpretive skills and techniques that allow for low false-positive rates, MRI should be further studied for its role as the primary imaging modality in breast screening and diagnosis.

## Introduction

Breast cancer is the most common malignancy for women in Taiwan [1]. The age-standardized incidence rate of female breast cancer increased rapidly from 39.6 per 100,000 in 2000 to 78.9 per 100,000 in 2017. In addition, higher breast density levels have been strongly associated with increased breast cancer risk, including Asian populations [2].

Screening for early breast cancer using mammography has been standard in many countries since the 1970s [3], based on mortality reductions seen in prospective, randomized trials, most notably the Swedish Two-County study [4]. Mammography used for diagnostic purposes emerged in tandem with screening, later incorporating breast ultrasound in the 1990s [5]. Subsequently, ultrasound emerged as a screening tool in its own right, primarily for women with dense tissue on mammography, with or without other risk factors [6].

When breast MRI became more widespread clinically in the early 2000s, it was used for surgical planning, diagnostic indications, and some screening applications, although it was not until 2007 that the first guidelines for MRI high-risk screening were introduced by the American Cancer Society [7]. Breast MRI is usually considered to be a second-tier imaging modality despite the fact that MRI sensitivity for breast cancer detection has consistently demonstrated a two-three fold cancer detection rate (CDR) compared to mammography [8]. This difference persists even when comparing MRI vs. 3D tomosynthesis and is particularly notable in women with dense breasts for whom the ability of mammographic screening to reduce mortality is debated [9].

A major drawback to wider utilization of breast MRI, beyond cost and convenience, is the reported low specificity, calculated at 68% in the American College of Radiology Imaging Network (ACRIN) 6883 trial [10]. With improved technology and greater experience, an MRI system designed and used only for breast imaging demonstrated a specificity of 89% in a multi-site trial that included both diagnostic and screening studies in 934 patients (95% specificity for screening; 84% specificity in diagnostic cases) [11]. While the false-positive rate (FPR) with breast MRI has been reported to be as high as 41% [12], the aforementioned multi-site trial involving 934 patients reported a false-positive rate of 11.2% for all cases, and only 4.9% for screening patients [11].

With these improvements in performance characteristics, the question arises as to whether or not breast MRI could be used as the primary modality for both screening and diagnosis. In this retrospective analysis of a regional breast screening program implemented by Taitung St. Mary’s Hospital, in Taiwan from 2013 to 2019, our goal was to evaluate the false-positive rate (FPR) and positive predictive value (PPV) associated with breast MRI when used as the primary breast imaging modality. Although conventional imaging was used in some cases for diagnostic clarification subsequent to the MRI, this study is unique in that a large cohort underwent MRI as the first step in asymptomatic screening, or for the initial workup of new symptoms or signs of breast disease.

## Materials and methods

The breast MRI program at Taitung St. Mary's Hospital was started in June 2013. Participants were women who registered as citizens or worked in Taitung and Hualien counties - two counties with high cancer mortality rates in Taiwan and a relative lack of healthcare resources [13]. Dedicated breast MRI was available free of charge for asymptomatic screening, or the workup of new symptoms or signs of breast disease. Additionally, before any breast imaging, health education lectures were provided for the participants to raise their awareness and understanding about breast cancer.

Technique of breast-dedicated MRI

All breast studies were performed on a 1.5 T dedicated breast MR system (Aurora Health US Corp, Danvers, USA) using a rotating delivery of excitation off resonance (RODEO) non-spoiled T1- and T2-weighted fat-suppressed sequence, followed by four spoiled T1-weighted data acquisitions at 1.5, 3.0, 4.5 and 6 minutes post-contrast injection. The slice display resolution was 512 x 512 at 700 microns width. Sequences were performed preceding and after the infusion of 0.1 mmol/kg gadolinium administered as a bolus dose with a power injector followed by 20 ml saline flush. Subtraction images were also performed. Interpretations were performed by radiologists with experience in all aspects of breast MRI.

Inclusion and exclusion criteria

We included women aged 20 years or older who underwent breast MRI as the primary imaging tool for initial screening or diagnosis at Taitung St. Mary's Hospital from 2013 to 2019. Patients were excluded if they had had a prior mammography or breast sonography within two years of their enrollment in the Taitung study. Also excluded were those who had a breast biopsy without imaging prior to their referral for participation, including those already diagnosed with cancer (breast imaging-reporting and data system {BIRADS} 6 classification).

This study was approved by the Kaohsiung V.G.H. Institutional Review Board (IRB number: KSVGH20-CT10-06). Since this was a retrospective study, informed consent was waived.

Data analysis

Performance characteristics were analyzed with a focus on cancer detection rate (CDR), positive predictive value (PPV), and FPR for the entire group. Because the presence or absence of pre-imaging symptoms was incompletely documented, screening and diagnostic studies were combined for analysis. Also, as this was a retrospective, observational study without a control group, or a second imaging method understudy, no conclusions could be made regarding superior Sensitivity or Specificity of one imaging method over another without a known false-negative rate. Thus, the focus of this large cohort was on the feasibility of breast MRI as the primary imaging tool, with regard to MRI-generated CDR, FPR, and PPV.

In assessing call-back rates and biopsies, FPR-1 represents any call-back for further imaging, FPR-2 represents call-backs that result in a biopsy recommendation, and FPR-3 represents call-backs that culminate in the actual performance of a (benign) biopsy. PPV-1, PPV-2, and PPV-3 are the correlates to the false positives when expressed as the percentage of biopsies that proved to be malignant. For purposes here, our focus was on FPR-3 and PPV-3.

Descriptive data included the age and breast density of the participants. Biomarker information from biopsy reports for MRI-detected cancers was collected and reported with regard to invasive disease vs. ductal carcinoma in situ (DCIS), and estrogen receptor (ER), progesterone receptor (PR), and human epidermal growth factor receptor-2 (HER2)neu status where available. Additionally, t-tests were applied for comparison between different age and breast density groups. All data were analyzed using Statistical Package for the Social Sciences (SPSS) version 22.0 (Armonk, NY: IBM Corp.).

## Results

From June 2013 to December 2019, 10,374 breast MRIs were performed in 7967 individuals at Taitung St. Mary’s Hospital, Taiwan. After excluding 2322 patients where the MRI was preceded by mammography or ultrasound, as well as 24 patients below the age of 20 years and two patients with BIRADS 6 classification, there were 5619 patients in whom the breast MRI was performed as the primary imaging modality for screening or diagnosis, forming the basis of this study.

Dedicated breast MRI identified 64 malignancies out of 5619 patients which corresponds to a CDR of 11 breast cancers detected per 1000 examinations. Among the 64 malignancies, 48 (75%) were invasive cancer and 16 (25%) were ductal carcinoma in situ (DCIS). Biomarker information, including ER, PR, and HER2neu for the malignant lesions are presented in Table 1. There was one triple-negative breast cancer case.

**Table 1:** Biomarker information of 64 cases with malignant findings by dedicated breast MRI Estrogen receptor (ER); progesterone receptor (PR); human epidermal growth factor receptor-2 (HER2)neu; ductal carcinoma in situ (DCIS)

	Invasive cancer (N=48)	DCIS (N=16)
	n (%)	n (%)
ER-positive	41 (85.4)	10 (62.5)
PR-positive	38 (79.2)	10 (62.5)
HER2neu-positive	19 (39.6)	6 (37.5)
Triple-negative	1 (2.1)	-

A total of 3761 (66.9%) of the subjects were under the age of 50 years, while 1858 (33.1%) were the age of 50 years or over. Demographics regarding age and breast density are presented in Table 2.

**Table 2:** Age and MRI breast density characteristics of the study patients *p < 0.001. **p < 0.01. False-positive rate (FPR-3); positive predictive value (PPV)

	Total participants (N=5619)	Total biopsies (N=415)	Malignant biopsies (N=64)	Benign biopsies (N=351)	FPR-3	PPV
	n (%)	n (%)	n (%)	n (%)	%	%
Age						
Under 50	3761 (66.9)	317 (76.4)	29 (45.3)	288 (82.1)	7.7*	9.1*
50 or over	1858 (33.1)	98 (23.6)	35 (54.7)	63 (17.9)	3.5	35.7
Breast density						
Level A	247 (4.4)	6 (1.4)	4 (6.3)	2 (0.6)	0.8*	66.7**
Level B	996 (17.7)	51 (12.3)	11 (17.2)	40 (11.4)	4.1	21.6
Level C	3766 (67.0)	315 (75.9)	43 (67.2)	272 (77.5)	7.3	13.7
Level D	596 (10.6)	41 (9.9)	4 (6.3)	37 (10.5)	6.3	9.8
Not specified	14 (0.2)	2 (0.5)	2 (3.1)	-		

Breast density was assigned by MRI criteria, with four (6.3%) of the malignancies identified in level A density patients, 11 (17.2%) in level B, 43 (67.2%) in level C, and four (6.3%) in level D, with two (3.1%) not specified. This distribution was comparable to the overall cohort where 247 (4.4%) of the 5619 were level A, 996 (17.7%) were level B, 3766 (67.0%) were level C, 596 (10.6%) were level D, while 14 (0.2%) were not specified (Table 2).

Sensitivity for MRI was nominally 100% by virtue of dedicated breast MRI serving as the referent. There was no long-term follow-up or secondary imaging performed concurrently, which would have allowed for the assessment of the false-negative rate. Without knowing the false-negative rate, neither sensitivity nor specificity can be calculated; however, several parameters that reflect specificity can be addressed, i.e., short-interval follow-up recommendation rate (BIRADS 3), false-positive rates, and positive predictive value.

Breast MRI prompted short-interval follow-up (BIRADS 3) in 974 (17.3%) studies out of 5619 with no biopsy required after the six-month follow-up period. An additional 142 (2.5%) patients who were scored originally as BIRADS 3 required a biopsy within six months of the MRI.

There were 351 benign biopsies performed out of the total cohort (n=5555 discounting malignant cases) for a false-positive rate (FPR-3) of 6.3%. Within the benign biopsies, there were 15 (4.3%) showing atypical hyperplasia, considered a clinically actionable high-risk lesion. Positive predictive value (PPV-3) was 15.4%, based on 64 true positives and 351 false positives.

Subgroup analysis revealed a statistically significantly lower false-positive rate and better PPV in women over 50 (Table 2). Statistical significance was also seen with low-density mammograms wherein false positives increased in proportion to breast density and PPV decreased with increasing density, both parameters negatively impacted by higher density.

## Discussion

Multi-modality imaging has become the standard of care in the screening and diagnosis of breast cancer. Mammography and ultrasound have long been considered the conventional approach, although contrast-enhanced MRI is now an integral part of the standard multi-modality assessment, having been introduced clinically nearly two decades ago.

Sensitivity for cancer detection using breast MRI exceeds mammography as well as ultrasound [8]. For women with dense breasts and risk factors, breast MRI sensitivity exceeds that of mammography and ultrasound combined [6].

High MRI sensitivity, with few missed cancers, comes at the cost of false-positive outcomes, hampering feasibility, both with regard to cost and other negative impacts of overdiagnosis. This was a particular problem shortly after the clinical introduction of breast MRI, with false-positive rates being reported at 32.2%, 35%, and 41% in three studies [9,11,14].

However, with increasing experience, a multi-site study using breast-dedicated MRI revealed a false-positive rate of 11.2% overall, with only a 4.9% false-positives rate with asymptomatic screening [10]. In this same study that included both screening and diagnostic cases, specificity was determined to be 88.8%, while sensitivity was 92%. The negative predictive value (NPV) in the screening population was 100% (326 of 326), and the area under the receiver operating characteristic (ROC) curve was 0.942.

With performance characteristics of dedicated breast MRI improving over time, the feasibility of using MRI as the primary imaging tool for breast cancer screening or diagnosis becomes more plausible. With the current multi-modality approach, the downside risks can be addictive, that is, using three modalities generates three cumulative false-positive rates. In this study, we sought to determine the false-positive rate and PPV when MRI is used as the initial study, rather than as a secondary or tertiary modality.

Understanding practical obstacles to using MRI upfronts, such as cost and efficient patient flow, there are technical improvements, some still under study, that could allow MRI to emerge as the primary modality for screening and/or diagnosis. This change in priority mandates low false-positive rates, as seen in this retrospective review of what may be the largest MRI series published to date.

There were some limitations in our study. First of all, we analyzed the initial biopsy results without the final (surgical) pathology available. Based on resources at Taitung St. Mary Hospital, participants who underwent dedicated breast MRI could have the initial biopsy performed locally. However, if the result was malignant, patients were referred to a higher-level hospital for further diagnostic studies and treatment. The results of their definitive treatment, if any, were not routinely available in the patient’s medical record at St. Mary’s. Secondly, the medical record often lacked detailed patient histories in this large cohort that would have allowed us to analyze screening data separately from diagnostic studies. Most commonly, it was unclear whether or not a breast complaint was related to what eventually proved to be cancer.

Another area where additional information would have been helpful was mammographic breast density. In our analysis, we used the MRI background density as reported, which is not necessarily the same as mammographic density [15]. That said, mammography was not commonly utilized after the MRI, so we elected to focus on the reported density level as judged by MRI, wherein patients were heavily skewed to lC (68.7%), as noted in Table 2. Since the overall distribution of patients among the four levels of MRI density was comparable to the density distribution in cancer patients, MRI density did not appear to play a major role in detection rates.

A variable that might have affected outcomes was the skewing of our cohort toward younger patients, with 66.9% of participants under the age of 50 years. For those patients who entered the study for asymptomatic screening purposes, this skewing toward younger patients would likely be associated with a lower rate of cancer incidence.

Simultaneously, this skewing toward younger patients was associated with a higher rate of false positives in the under-50 group. The 7.7% false-positive rate in this younger group, even though statistically significant compared to the age 50 years and above (3.5%), is still quite low compared to the earlier breast MRI studies. In fact, the most significant finding in our study of MRI as a primary imaging tool was the very low false-positive rate for our entire cohort (6.3%). PPV-3 (15.4%) for the cohort was at the lower end of an expected range from 12% to 30% [16]. This is likely a reflection of the cohort being skewed toward the younger age group (66.9% of participants under the age 50 years) wherein PPV was 9.1% compared to 35.7% PPV-3 in the 50 years and older group.

While two major MRI screening studies have used MRI as the only imaging tool - one designed for average-risk patients and one for women with level D mammographic density - entry to both those studies required negative mammograms, and for the former, negative screening ultrasound in most cases (65%) as well [17,18]. To our knowledge, this present study represents the first time breast MRI has been used as the primary imaging tool in both a screening and diagnostic setting.

## Conclusions

Utilizing breast MRI as the initial study for screening and/or diagnosis appears to be limited more by practical considerations such as cost and patient flow efficiency than by feasibility based on performance characteristics. 

In this study where MRI identified 64 malignancies from 5619 MRI scans (1.1%), the false-positive rate (FPR-3) of 6.3% and positive predictive value (PPV-3) of 15.4% support the feasibility of breast MRI. Additionally, when considering that the population was skewed to the under 50 age group, performance characteristics are quite acceptable.
